# Control of Trachoma in Australia: A Model Based Evaluation of Current Interventions

**DOI:** 10.1371/journal.pntd.0003474

**Published:** 2015-04-10

**Authors:** Andrew J. Shattock, Manoj Gambhir, Hugh R. Taylor, Carleigh S. Cowling, John M. Kaldor, David P. Wilson

**Affiliations:** 1 The Kirby Institute, University of New South Wales, Sydney, Australia; 2 Department of Epidemiology and Preventive Medicine, Monash University, Melbourne, Australia; 3 Melbourne School of Population Health, The University of Melbourne, Melbourne, Australia; University of Cambridge, UNITED KINGDOM

## Abstract

**Background:**

Australia is the only high-income country in which endemic trachoma persists. In response, the Australian Government has recently invested heavily towards the nationwide control of the disease.

**Methodology/Principal Findings:**

A novel simulation model was developed to reflect the trachoma epidemic in Australian Aboriginal communities. The model, which incorporates demographic, migration, mixing, and biological heterogeneities, was used to evaluate recent intervention measures against counterfactual past scenarios, and also to assess the potential impact of a series of hypothesized future intervention measures relative to the current national strategy and intensity. The model simulations indicate that, under the current intervention strategy and intensity, the likelihood of controlling trachoma to less than 5% prevalence among 5–9 year-old children in hyperendemic communities by 2020 is 31% (19%–43%). By shifting intervention priorities such that large increases in the facial cleanliness of children are observed, this likelihood of controlling trachoma in hyperendemic communities is increased to 64% (53%–76%). The most effective intervention strategy incorporated large-scale antibiotic distribution programs whilst attaining ambitious yet feasible screening, treatment, facial cleanliness and housing construction targets. Accordingly, the estimated likelihood of controlling trachoma in these communities is increased to 86% (76%–95%).

**Conclusions/Significance:**

Maintaining the current intervention strategy and intensity is unlikely to be sufficient to control trachoma across Australia by 2020. However, by shifting the intervention strategy and increasing intensity, the likelihood of controlling trachoma nationwide can be significantly increased.

## Author Summary

Australia is the only remaining high-income country reporting endemic levels of trachoma, with infections occurring predominantly within rural and remote Indigenous communities. Although the Australian government has recently invested large sums of money to combat the disease, it remains unclear whether the national goal of controlling trachoma by 2020 will be achieved. Here, we use a novel individual-based simulation model to estimate the impact of numerous potential future invention strategies and intensities. Our model is the most sophisticated trachoma transmission model to date, and the first to specifically represent trachoma in Australian Indigenous communities. Model projections suggest that although the current intervention strategy and intensity are unlikely to achieve the target of national control by 2020, the likelihood of achieving this goal can be significantly increased by shifting the intervention strategy and increasing the intensity of key intervention components such as screening, treatment and facial cleanliness activities. Our findings that the most resource rich country with endemic trachoma may require a more intensive intervention effort to control the disease suggest that challenges may remain in the fight for the global control and eventual elimination and eradication of trachoma.

## Introduction

Australia is the only high-income country in which trachoma, the worldwide leading cause of preventable blindness [1], remains endemic [2]. In remote Aboriginal communities deemed to be at-risk of trachoma, an estimated 4% of adults suffer severely impaired vision or blindness [3] due to many years of repeated re-infection with the bacterium *Chlamydia trachomatis*—the infectious agent from which trachoma disease develops [4]. In 2009, the Australian government pledged AUS$16 million over an initial four-year period towards the national goal of controlling trachoma by 2020 [3]. That is, to reduce the prevalence of *trachomatous inflammation follicular* (TF) to less than 5% amongst 5–9 year-old children within a community. This target closely aligns with the Global Elimination of Trachoma by 2020 (GET 2020) initiative [5] developed by the World Health Organisation (WHO). The Australian trachoma intervention effort combines annual surveillance activities with a *Surgery*, *Antibiotics*, *Facial cleanliness and Environmental improvement* (SAFE) control policy recommended by the WHO [3,6]. This four-component policy incorporates treatment for those with clinically detected disease and long-term solutions for reducing infection incidence and disease prevalence [7]. The WHO offers recommendations for the frequency and intensity of the screening and treatment programs integrated into the SAFE policy [8]; however, the Australian intervention effort involves a greater intensity of screening and treatment due to larger resource availability compared to other trachoma-endemic countries. Despite this, the prevalence of trachoma remains high in many Aboriginal communities [3] whilst several developing countries prepare to announce the national control or eradication of the disease [9].

In this paper, we assess the progress of recent trachoma intervention efforts in Australia and evaluate the possibility of achieving national control by 2020. This is achieved by addressing the following three questions: (i) have past trachoma intervention efforts been effective in reducing infection incidence and disease prevalence? (ii) what epidemiological impact can be expected if the current intervention strategy and intensity is maintained until 2020? (iii) how can a shift in strategy or increase in intensity improve this impact? These questions are addressed through the development and analysis of a novel simulation model of trachoma transmission in remote Australia.

Previous models of trachoma transmission have typically implemented population-based methods [10–14]. These traditional models can be useful for extracting general principles but often lead to an over-simplification of disease dynamics [15]. Recent studies have indicated that transmission between two individuals is influenced by factors such as age, with children younger than 10 years being the typical reservoir of infection [12,16], and the presence of nasal or ocular discharge, i.e. a dirty face [17]. Demographic factors such as household overcrowding [10,11] and inter-community migration are also believed to contribute to trachoma persistence [18]. The temporary migration of individuals between communities is believed to be of particular importance in sustaining endemic trachoma in Australia [18]. Here, an individual-based simulation model is developed to incorporate these complexities. This is the most sophisticated trachoma transmission model to date, and the first model to specifically represent endemic trachoma in Australian Aboriginal communities. The parameters of the model are informed by the best available Australian and international data (see S1 Table).

## Methods

**Fig 1:**
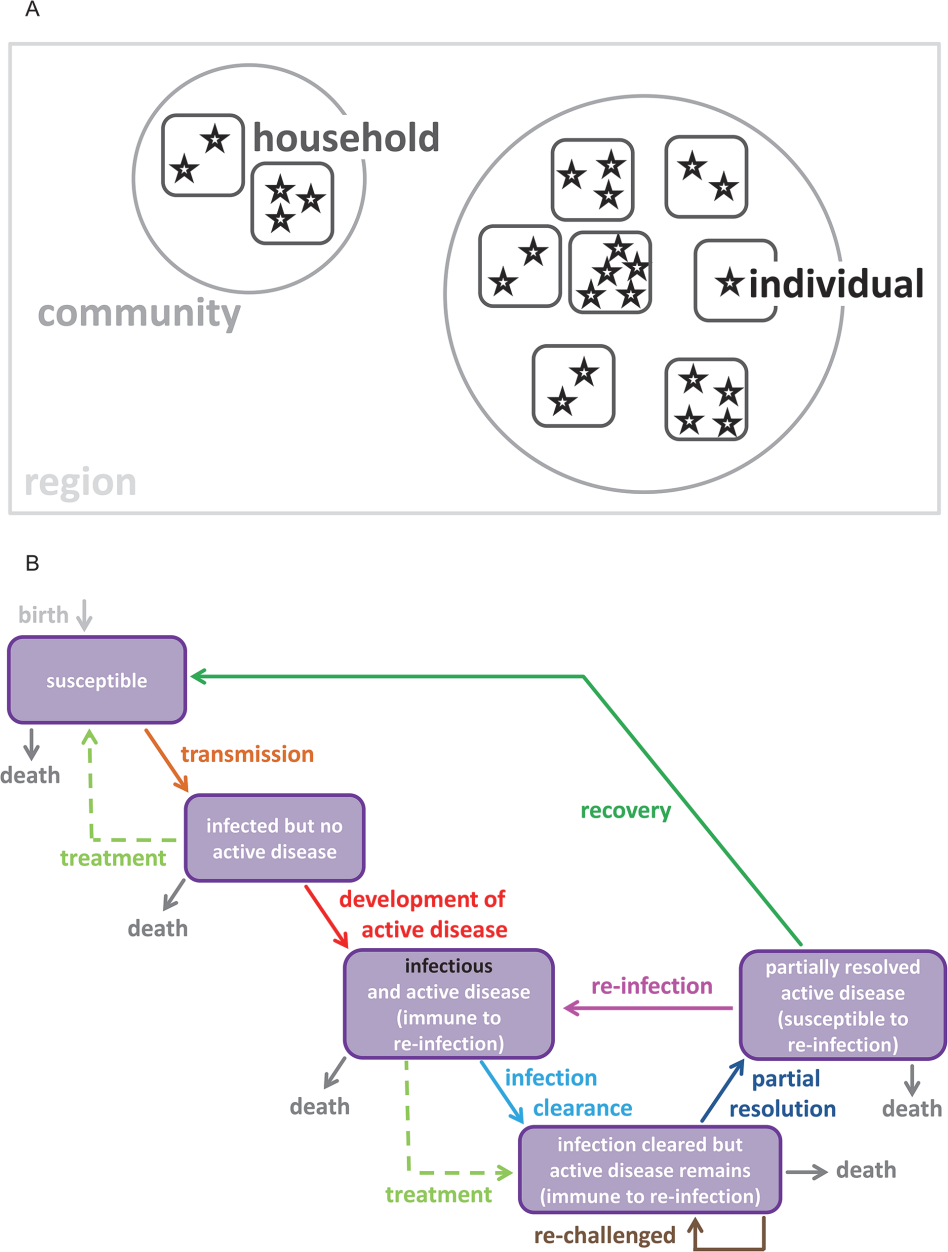
(a) Illustration of the model structure. The stars represent individuals, the rounded squares represent households, the circles represent communities and the rectangle represents a region. Each individual has a propensity to temporarily migrate from their usual abode in their home community to a household in a neighbouring community. Transmission can occur between individuals residing (either permanently or temporarily) within a household or between individuals currently within a community. Age-stratified mixing patterns differ dependent upon whether individuals are of the same household, a different household in the same community, or are temporary visitors to the household or community. **(b)** Natural history of trachoma utilised by the model. After contracting infection an individual enters a short latent period where infection load is such that the newly infected individual is not yet infectious, whilst active disease has yet to develop. The natural immune response then develops and the infected individual progresses to an infectious state where clinical disease is evident. Whilst in this stage of disease progression, the individual is not susceptible to re-infection. Following the clearance of infection, the individual enters a disease-only state in which they are immune to re-infection, but are subject to a prolonged disease episode when re-challenged. After partially resolving the clinical disease, an individual progresses into a second disease-only state where they are susceptible to re-infection. In the event that no re-infection occurs during this episode, the individual fully recovers to the susceptible state. The duration of each infection and disease state is dependent upon the age of the individual.

The model developed for this study simulates a population of Aboriginal persons within a remote Australian region. Each individual represented in the model is a member of an at-risk community encompassed by the region, and is also a resident of a household within a community (Fig 1A). The temporary migration of individuals (and potentially other members of their household) is simulated based on rates of movement between communities [19].

The model is characterised by a five-state natural history structure (Fig 1B). Upon infection with *C*. *trachomatis* an individual enters a short latent period where infection load is such that the newly infected individual is not yet infectious, whilst active disease (*trachomatous inflammation follicular/intense*: TF/TI) has yet to develop [20]. An immunopathological response then develops and the infected individual progresses to an infectious state where clinical disease appears [4]. Following the clearance of infection, the inflammatory disease state resolves slowly in the absence of reinfection. Individuals in this state are partially immune to re-infection, but if re-challenged will experience prolonged disease [21]. In the event that no re-infection occurs during this episode, the individual fully recovers to the susceptible state. The duration of each infection and disease state is dependent upon exposure to repeated episodes of infection. Exposure to reinfection is assumed to decrease with age[16].

The transmission of infection and the subsequent development of disease are stochastically determined at the individual-level [22]. That is, the probability of infection transmission between an infectious individual and a susceptible individual is calculated and a random number generated to determine whether transmission will occur at the relevant time point. The probability of transmission between two individuals is assumed to be influenced by the ‘clean face’ status of both the susceptible individual and the infectious individual, where facial cleanliness is assumed to reduce the probability of transmitting and contracting infection [17]. Age-stratified community-level facial cleanliness prevalence data has been recorded across remote Australia as a process of trachoma screening events since 2007, and is directly entered into the model [23]. The probability of transmission is also affected by the infectiousness of the infected individual, assumed to be proportional to the bacterial load, which in-turn is assumed to be dependent upon the number of previous infections [12]. Two distinct settings for human interaction, and thus transmission potential, are considered in the model. The primary setting for transmission is the household, although transmission can also occur in the wider community. See S1 Text for further details and model equations.

The model described was independently calibrated to empirical age-stratified community-level disease prevalence data from three Australian regions through first-order Monte Carlo filtering methods [24]. See S1 Table and S1 File for model parameters and further details of the model and the calibration process. The model source code is also available on-line [25]. Each modelled community was classified as *hyperendemic* (≥ 20% active trachoma disease prevalence in 5–9 year olds), *mesoendemic* (≥ 10% but < 20%) or *hypoendemic* (≥ 5% but < 10%) dependent on the mean community disease prevalence observed from 2007 to 2011. The three modelled regions were selected to form a representative sample of trachoma-endemic remote Australia, with selection based on the endemicity of the communities within each region as well as the quantity and quality of surveillance data available. Throughout this paper, the simulated regions are de-identified and referred to as *‘predominantly hyperendemic’*, *‘predominantly mesoendemic’* and *‘predominantly hypoendemic’* based on the prevailing endemicity of the communities within the region. Communities with a consistent 5–9 year old disease prevalence of less than 5% are considered not-at-risk, with 5% also considered the threshold for control [8]. The timing and age-stratified intensity of 2007–2011 screening and treatment events were directly entered into the model according to programmatic monitoring data.

The calibrated model was utilised to evaluate the impact of recent intervention efforts. This was achieved by simulating the model in the absence of past intervention efforts such as screening programs, treatment events, housing development initiatives and improvements in facial cleanliness prevalence. A direct comparison was made between the model calibrated to reflect observed conditions and the model output under the hypothetical scenario of no past intervention efforts. The model was then used to project the future impact of a series of potential intervention scenarios. A base-case future intervention scenario was compiled by extrapolating the trends from previously observed trachoma intervention events. The values obtained through this analysis are presented in S2 Table, whilst a description of the current National Guidelines for Trachoma Control in Australia are presented within S3 Table. This base case scenario was then analysed against a series of alternative intervention scenarios. The results obtained from a selection of these alternative scenarios, which are described in Table 1, are presented in this paper.

**Table 1:** The shift in intervention strategy and intensity for the range of the alternative future intervention strategies considered.

Alternative future intervention scenario		Shift in strategy direction	Shift in intervention intensity attained
1	Increase housing construction.	None.	Annually construct 10% of community total number of houses.
2	Increase facial cleanliness.	None.	Implement facial cleanliness programs such that at least 70% of 1–4 year olds and at least 90% of 5–14 year olds consistently have clean faces.
3	Increase facial cleanliness, screening and treatment.	None.	Screen at least 70% of 1–4 year olds and at least 90% of 5–14 year olds, treat 98% of those requiring treatment within 2 weeks, and achieve the facial cleanliness targets described in alternative scenario 2.
4	Combination of all interventions.	Continuously implement MDA in hyperendemic communities every 6 months for 3 years before re-screening.	Achieve the targets described in alternative scenario 3.
5	Current targets.	None.	Screen at least 80% of 5–9 year olds, treat 90% of those requiring treatment within 2 weeks, and achieve a 70% facial cleanliness prevalence in 1–14 year olds.
6	Proposed targets.	See S3 Table.	See S3 Table.

Each of the future intervention scenarios were simulated until 2020 and the community-level age-stratified prevalence of infection and disease were recorded in each modelled community. The likelihood of controlling trachoma was then calculated as the proportion of model simulations, for each community, in which the control criterion was satisfied by 2020. To produce representative outputs which accounted for the stochasticity of the model, 1,000 simulations were produced using 1,000 distinct parameter sets. These parameter sets were sampled from the realistic range of plausible parameter estimates obtained through the model calibration process (described in S1 File). These results were aggregated to form control likelihood estimates for communities of specific endemicity under a given intervention scenario. All numerical computation was performed using MATLAB [26].

## Results

**Fig 2:**
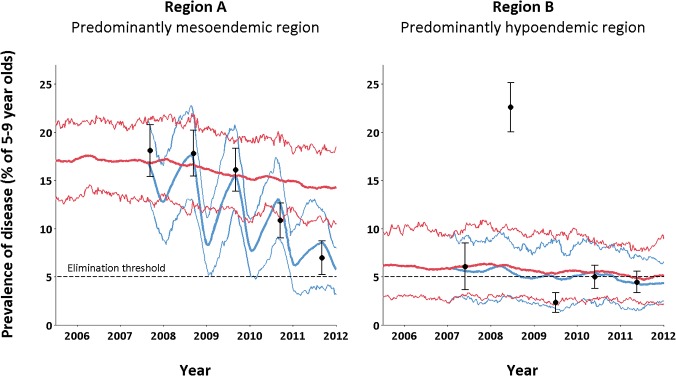
The epidemiological effect of past intervention efforts on disease prevalence amongst 5–9 year old children in two de-identified remote Australian regions. The thin blue curves represent the 90% inter-percentile range of 1,000 model simulations with the past intervention effort applied as empirically recorded. The thick blue curve illustrates the mean simulation. The corresponding red curves describe the hypothetical scenario that a trivial intervention effort was observed. The black dots represent the weighted disease prevalence levels of 5–9 year old children found through annual screening events in each of the at-risk communities modelled within the specific region. Accompanying each of the observed prevalence points is a 95% confidence interval which reflects the regional screening coverage achieved in the given year. The extreme 2008 prevalence in the right hand figure is likely due to the temporary mass migration of individuals [28].

Model-based evaluations of the interventions implemented between 2007 and 2011 suggest that disease prevalence has generally been reduced through trachoma intervention efforts. However, the scale of impact of the past intervention measures was found to vary between regions. The greatest reductions were observed in the predominantly hyperendemic regions, where trachoma prevalence among 5–9 year old children was estimated to have been 23.5% (mean from 1,000 simulations, with range 18.5%–30.7%) in 2011 in the absence of interventions compared with 14.3% (10.5%–18.5%) with interventions; in the predominantly mesoendemic region, trachoma prevalence was estimated to have reduced from 14.8% (10.3%–19.7%) to 5.8% (3.2%–8.0%) due to intervention efforts (Fig 2). However, the impact of intervention measures in the predominantly hypoendemic region is more modest: disease prevalence in 2011 was estimated to have reduced from 5.1% (2.2%–8.9%) to 4.3% (2.3%–6.5%) (Fig 2). This occurs despite a comparable, if not stronger, screening and treatment effort being observed in the hypoendemic region. Indeed, the mean 5–9 year old screening coverage from 2007 to 2011 in the predominantly mesoendemic and predominantly hypoendemic regions are 70.5% and 78.9%, respectively. The corresponding values for treatment coverage were 87.6% and 86.1%, respectively. A sensitivity analysis of model input parameters (see S1 File) suggests that this finding may be influenced by a higher baseline prevalence of child facial cleanliness in the predominantly hypoendemic region.

**Fig 3:**
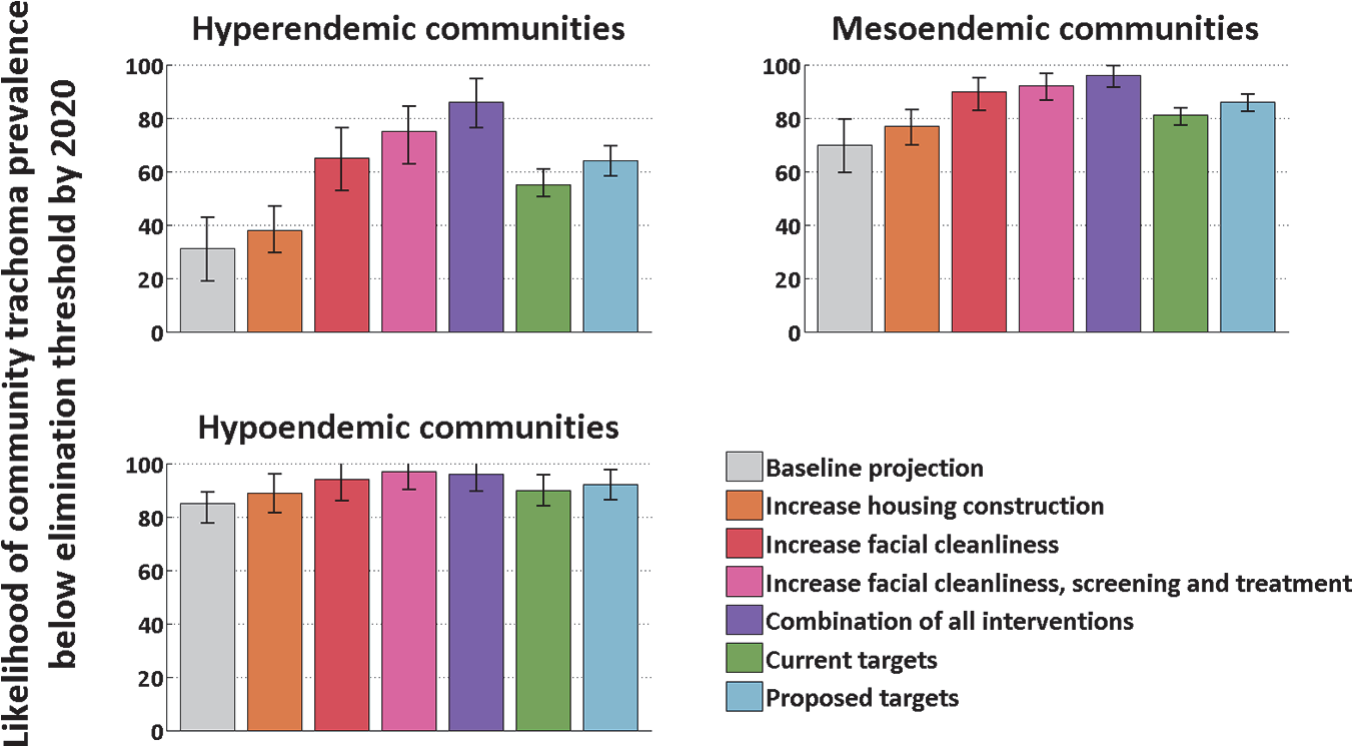
The aggregated likelihood of satisfying the trachoma control criteria by 2020 within a community under each of the considered future intervention scenarios, segregated by community endemicity. The values illustrated in the top left subplot indicate the significant increase in control likelihood that can be achieved by enhancing the intervention strategy and intensity in the worst affected communities.

Future projection simulations estimate the likelihood of achieving trachoma control in hypoendemic and mesoendemic communities by 2020 to be 85% (77%–89%) and 70% (60%–79%), respectively, should current trachoma intervention efforts be maintained (Fig 3). However, the likelihood of satisfying the trachoma control criteria in hyperendemic communities under this scenario was calculated to be only 31% (19%–43%). The estimated likelihoods of controlling trachoma in hypoendemic communities by 2020 were found to be consistently high across each of the considered intervention scenarios. However, large differences were found in control likelihoods in the mesoendemic and, in particular, hyperendemic communities across the modelled future scenarios. This suggests that by optimising the intervention strategy and intensity, the likelihood of achieving trachoma control in highly endemic communities can be greatly increased.

Increasing housing construction, and therefore easing the burden of household overcrowding, in addition to maintaining the current intervention strategy increased the estimated likelihood of achieving trachoma control in hyperendemic communities from 31% (19%–43%) to 38% (30%–47%). Alternatively, assuming a 1.5-2-fold reduction in both infectiousness and susceptibility due to facial cleanliness[18], achieving a consistently high facial cleanliness prevalence (90%) amongst children was found to increase the likelihood of controlling trachoma from these worst-affected communities by 2020 to 64% (53%–76%). By additionally attaining consistently large screening and treatment coverages, this likelihood of control was estimated to be 75% (63%–85%). The epidemiological effect of this combination of interventions was found to be greater than the sum of the individual interventions, suggesting that a synergistic effect exists between screening, treatment and attaining high levels of facial cleanliness amongst children. The greatest likelihood for achieving control in the worst-affected communities occurred when these intervention intensities were further coupled with alterations in the treatment strategy; by introducing bi-annual mass drug administration (MDA) in hyperendemic communities, the likelihood of achieving trachoma control increased to 86% (76%–95%), with a corresponding control likelihood of 96% (92%–100%) in mesoendemic communities. This most-effective future scenario was projected out until 2030, and no rebounding of the epidemic was observed.

**Fig 4:**
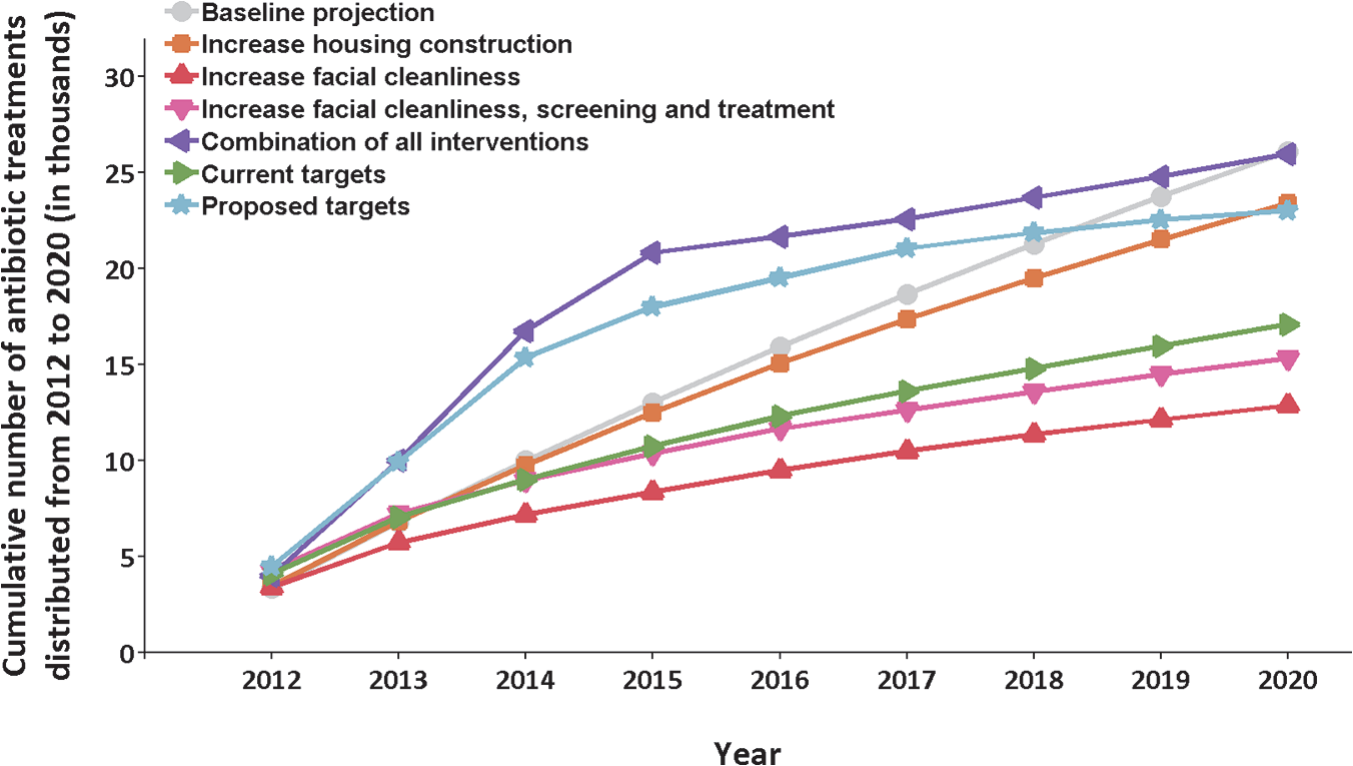
The cumulative number of antibiotic treatments distributed in the predominantly hyperendemic region under a selection of the future intervention scenarios between 2012 and 2020. The reduced number of antibiotics required under the ‘increase facial cleanliness’, ‘increase facial cleanliness, screening and treatment’, and ‘current targets’ future scenarios reflects the lower incidence rates observed when the young population has a higher clean face prevalence. This lower incidence results in a lower disease prevalence and therefore fewer communities require large scale antibiotic programs to control the disease. The ‘proposed targets’ and ‘combination of all interventions’ scenarios illustrate the sharp initial increase in antibiotic distribution required to implement policies in which repeated MDA is administered to hyperendemic communities. However, the antibiotic distribution effort required under these scenarios is drastically reduced following an early peak, demonstrating the effectiveness of such programs in drastically reducing disease prevalence.

The resources required to achieve control is an important consideration. Here, crude estimations of the resources required across the predominantly hyperendemic region were calculated for each scenario by the total number of people receiving treatment (Fig 4). An estimated total of 26,088 antibiotic doses are to be distributed between 2012 and 2020 under the current intervention strategy and intensity. This value compares with 12,855 treatments under the scenario in which ambitious yet feasible child facial cleanliness prevalence targets were also consistently satisfied. The significantly smaller number of treatments reflects the lower incidence rates attained when a substantially larger proportion of the young population had clean faces. Under the future scenario where ambitious yet feasible screening, treatment and facial cleanliness targets were consistently met, 15,312 antibiotic doses would be distributed. Despite this slight increase in antibiotic distribution compared with the previous scenario, the large increase in control likelihood that can be attained by implementing such a control policy and intensity, particularly in hyperendemic communities, makes a solid case for implementing such a control effort. A bi-annual MDA program would result in 25,989 antibiotic doses distributed. A large proportion of this total would be distributed within the first three years of implementing the strategy; however, the treatment effort required following this initial peak would decline over time to be less than that required under the current control strategy (Fig 4). The MDA strategy, with substantially greater control likelihood, emphasises the ‘hit hard, hit early’ principle for greatest effectiveness and cost-effectiveness.

## Discussion

Australia is the only high-income country to have endemic trachoma. Whilst being a signatory to the WHO’s GET 2020 initiative, the Australian government has responded to the health issue in recent years with large investment. However, as several developing countries with histories of trachoma prepare to announce the national control or eradication of the disease [9], high prevalence levels of trachoma are still observed in remote Australian Aboriginal communities. Since 2006, Australia has implemented national surveillance activities to collect age-segregated community-level data describing the timing, frequency and intensity of screening and treatment programs as well as disease prevalence, facial cleanliness prevalence, and more recently environmental conditions that may affect trachoma incidence and persistence [3,23,27]. Increases in community screening and treatment, along with recorded increases in facial cleanliness among children has correlated with declines in trachoma prevalence in Australia.

Although our model estimates that current strategies and intensities of programs are unlikely to lead to national control, alternate scenarios appear to be feasible and effective means of achieving this goal. Our results suggest that to achieve control in the worst-affected communities, a more intense intervention effort may be required. For hypoendemic communities (prevalence 5–10%), the model output indicates that continuing the current intervention strategy and intensity will likely be sufficient to control trachoma by 2020. By assuming that facial hygiene programs can reduce transmission potential over 2-fold, our model estimates that a substantial increase in community facial cleanliness prevalence may be sufficient to control trachoma by 2020 in mesoendemic communities (prevalence 10–20%). The model results indicate that annual screening events are appropriate in mesoendemic communities, whilst the long-term gain of implementing mass drug administration (MDA) as opposed to treating only the household contacts of index cases was found to have a negligible impact. The model predicts that the future intervention effort required to considerably raise the likelihood of achieving control in hyperendemic communities (prevalence >20%) by 2020 is more difficult. An increase in screening, treatment and facial cleanliness prevalence should be combined with an enhanced housing construction program. Continuous bi-annual MDA is also recommended for three consecutive years before resuming screening events to significantly raise the likelihood of controlling trachoma in these most endemic communities. However, it should also be noted that it may be possible for active disease amongst children to reach below 5% by 2020 even if all trachoma-specific interventions were discontinued immediately. Such decreases are not unusual in far less wealthy regions of the world where programmatic coverage has been poor. But without specific interventions in the past, the rates of trachoma have changed very little in these communities over many years. Thus, we believe concerted and targeted approaches—informed by this analysis—will increase the chance of trachoma control.

Previous models of trachoma transmission have described the natural history of trachoma infection and disease using simple population-based systems of differential equations [10–14]. Although useful for extracting general principles, population-based models are limited by the level of complexity that they are able to incorporate. In the context of trachoma control in Australia, the range of complexities one can consider—such as an individual’s age, facial cleanliness status, usual residence and the environmental state of their home community—lends itself to a more flexible model with finer granularity. As such, this study is based on a detailed individual-based simulation model, informed by and calibrated to relatively large amounts of data for remote Australian Aboriginal communities experiencing trachoma. The model developed here attempts to accurately represent the natural history of trachoma infection and disease whilst also assimilating the demographic, cultural and biological factors that influence ocular *C*. *trachomatis* transmission. Whilst no model may ever be sophisticated enough to capture all of the heterogeneities involved in the transmission of an infectious disease, the usefulness of the output hinges on the optimality of the model’s balance between complexity and accuracy [22]. Extensive collaboration was sought to ensure that the model described in this paper achieved such a balance. However, it is imperative that the results presented must be consumed with perspective.

Other limitations that should be addressed when assessing the validity of modelling results regard the empirical data that are used to inform the model parameters. For the purpose of this research, a large volume of nationally collated surveillance data was employed but these data are also potentially limited in completeness and representativeness. There exists a certain degree of uncertainty in the estimates of trachoma prevalence, particularly in the early years of data collection, as there was some degree of variation in screening coverage rates. However these coverages have progressively improved along with the accuracy of trachoma grading. Australian treatment coverage data can be difficult to interpret as the method of distribution has varied and has not always been clearly specified; these methods have included MDA, household contact based treatment and treating only affected children. Equally, the definition of the denominator and hence the coverage achieved have also varied somewhat. The model also assumes a steady-state equilibrium at baseline, which may be inaccurate as a result of previous trachoma treatment efforts. Although these are important limitations and have an impact on the precision of the forward estimates of effectiveness of the interventions, they do not influence the comparisons of the relative effectiveness of the different strategies. This is a strength of this research as it shows the greater effectiveness of a combined more intensive strategy compared with that currently employed.

The world has a goal of eliminating blinding trachoma as a public health concern. The countries which have done so or are on the verge of announcing such success should be commended for their excellent public health efforts. However, current strategies may not be sufficient in other contexts and as we have demonstrated in this study, they may be insufficient in the trachoma-endemic country with the greatest amount of resources. Through detailed simulation modelling we have suggested some slight shifts in strategies and changes in intensities in the short-term which have the potential to yield substantial returns in the future in order to achieve this ultimate goal.

## Supporting Information

S1 Table Model parameters. (DOCX)

S2 Table Extrapolating current intervention trends. (DOCX)

S3 Table Current and proposed guidelines. (DOCX)

S1 Text Underlying model assumptions. (DOCX)

S1 File Calibration process and sensitivity analysis. (DOCX)

## References

[1] Burton MJ, Frick KD, Bailey RL, Bowman RJC (2002) Azithromycin for the treatment and control of trachoma. Expert Opin Pharmacother 3: 113–120. 11829725 10.1517/14656566.3.2.113

[2] Taylor HR, Fox SS, Xie J, Dunn RA, Arnold A-LMR, et al (2010) The prevalence of trachoma in Australia: the National Indigenous Eye Health Survey. Med J Aust 192: 248–253. 20201757 10.5694/j.1326-5377.2010.tb03501.x

[3] Cowling CS, Popovic G, Liu BC, Ward JS, Snelling TL, et al (2012) Australian Trachoma Surveillance Annual Report, 2010. Communicable Diseases Intelligence 36. 10.33321/cdi.2012.36.18 23186235

[4] Grassly NC, Ward ME, Ferris S, Mabey DC, Bailey RL (2008) The natural history of trachoma infection and disease in a Gambian cohort with frequent follow-up. PLoS Negl Trop Dis 2: e341 10.1371/journal.pntd.0000341 19048024 PMC2584235

[5] World Health Organization (2004) Report of the Eighth Meeting of the WHO Alliance for the Global Elimination of Blinding Trachoma. Geneva.

[6] Wright HR, Keeffe JE, Taylor HR (2010) Barriers to the implementation of the SAFE strategy to combat hyperendemic trachoma in Australia. Ophthalmic Epidemiology 17: 349–359. 10.3109/09286586.2010.528135 21090909

[7] West SK (2003) Blinding trachoma: prevention with the safe strategy. Am J Trop Med Hyg 69: 18–23. 14692676 10.4269/ajtmh.2003.69.18

[8] Solomon AW, Zondervan M, Kuper H, Buchan JC, Mabey DCW, et al (2006) Trachoma control: a guide for programme managers: World Health Organization.

[9] World Health Organization (2012) Report of the Sixteenth Meeting of the WHO Alliance for the Elimination of Blinding Trachoma by 2020. Washington DC.

[10] Blake IM, Burton MJ, Solomon AW, West SK, Basanez M-G, et al (2010) Targeting antibiotics to households for trachoma control. PLoS Negl Trop Dis 4: e862 10.1371/journal.pntd.0000862 21072225 PMC2970531

[11] Blake IM, Burton MJ, Bailey RL, Solomon AW, West S, et al (2009) Estimating household and community transmission of ocular Chlamydia trachomatis. PLoS Negl Trop Dis 3: e401 10.1371/journal.pntd.0000401 19333364 PMC2655714

[12] Gambhir M, Basanez M-G, Burton MJ, Solomon AW, Bailey RL, et al (2009) The development of an age-structured model for trachoma transmission dynamics, pathogenesis and control. PLoS Negl Trop Dis 3: e462 10.1371/journal.pntd.0000462 19529762 PMC2691478

[13] Ray K, Lietman T, Porco T, Keenan J, Bailey R, et al (2009) When can antibiotic treatments for trachoma be discontinued? Graduating communities in three African countries. PLoS Negl Trop Dis 3: e458 10.1371/journal.pntd.0000458 19529761 PMC2690652

[14] Ray KJ, Porco TC, Hong KC, Lee DC, Alemayehu W, et al (2007) A rationale for continuing mass antibiotic distributions for trachoma. BMC Infect Dis 7: 91 17683646 10.1186/1471-2334-7-91 PMC1988814

[15] Gambhir M, Basanez M-G, Blake IM, Grassly NC (2010) Modelling trachoma for control programmes. Adv Exp Med Biol 673: 141–156. 20632535 10.1007/978-1-4419-6064-1_10

[16] Bailey R, Duong T, Carpenter R, Whittle H, Mabey D (1999) The duration of human ocular Chlamydia trachomatis infection is age dependent. Epidemiology and Infection 123: 479–486. 10694161 10.1017/s0950268899003076 PMC2810784

[17] Lansingh VC, Mukesh BN, Keeffe JE, Taylor HR (2010) Trachoma control in two Central Australian Aboriginal communities: a case study. International Ophthalmology 30: 367–375. 10.1007/s10792-010-9360-5 20358257

[18] Taylor HR (2008) Trachoma: A blinding scourge from the bronze age to the twenty-first century: Centre for Eye Research Australia.

[19] Biddle N, Prout S (2009) The geography and demography of Indigenous temporary mobility: an analysis of the 2006 census snapshot. Journal of Population Research 26: 305–326.

[20] Jawetz E, Rose L, Hanna L, Thygeson P (1965) Experimental inclusion conjunctivitis in man: measurements of infectivity and resistance. JAMA 194: 620–632. 5319187

[21] Taylor HR, Johnson SL, Prendergast RA, Schachter J, Dawson CR, et al (1982) An animal model of trachoma II. The importance of repeated reinfection. Invest Ophthalmol Vis Sci 23: 507–515. 6749750

[22] Keeling MJ, Rohani P (2007) Modeling Infectious Diseases in Humans and Animals: Princeton University Press.

[23] Tellis B (2008) Trachoma Surveillance Report 2007: National Trachoma Surveillance and Reporting Unit. Centre for Eye Research Australia.

[24] Groot Koerkamp B, Weinstein MC, Stijnen T, Heijenbrok-Kal MH, Hunink MG (2010) Uncertainty and patient heterogeneity in medical decision models. Med Decis Making 30: 194–205. 10.1177/0272989X09342277 20190188

[25] Shattock AJ (2014) Trachoma- Modelling. http://figshare.com/articles/Trachoma_Modelling/1164206 (accessed 2014/09/08).

[26] MATLAB version 7.13.0 Natick, Massachusetts The MathWorks Inc., 2011.

[27] Tellis B (2009) Trachoma Surveillance Report 2008: National Trachoma Surveillance and Reporting Unit. Centre for Eye Research Australia.

[28] Department of Families H, Community Services and Indigenous Affairs (2012) Northern Territory Emergency Response. http://www.fahcsia.gov.au/our-responsibilities/indigenous-australians/programs-services/closing-the-gap/closing-the-gap-engagement-and-partnership-with-indigenous-people/northern-territory-emergency-response: Australian Government.

