# Supplementary material for: Control of Trachoma in Australia: A Model Based Evaluation of Current Interventions
Source: PLoS Negl Trop Dis. 2015 Apr 10;9(4):e0003474. doi: 10.1371/journal.pntd.0003474 (PMC4393231; doi:10.1371/journal.pntd.0003474)
Supplement: S1 Table — (DOCX) [file pntd.0003474.s001.docx]

S2 Table. Model parameters.

**Demographic parameters**

| Number of modelled ‘at-risk’ communities | | 67 | [^1-5^](#_ENREF_1) |
| --- | --- | --- | --- |
| Number of houses in modelled communities in 2006 | | 4,789 | [^6^](#_ENREF_6) |
| Number of modelled 0-4 year olds in 2006 | | 2,746 | [^6^](#_ENREF_6) |
| Number of modelled 5-9 year olds in 2006 | | 2,898 | [^6^](#_ENREF_6) |
| Number of modelled 10-14 year olds in 2006 | | 2,540 | [^6^](#_ENREF_6) |
| Number of adults (15+ year olds) in 2006 | | 14,901 | [^6^](#_ENREF_6) |
| Total number of modelled individuals in 2006 | | 23,085 | [^6^](#_ENREF_6) |
| Birth rate (births per 1,000 resident population) | | 17.0 | [^7^](#_ENREF_7) |
| Death rate | Under 12 months old (deaths per 1,000 live births) | 13.1 | [^8^](#_ENREF_8) |
|  | 1-4 year olds (deaths per 100,000 population) | 69.6 | [^8^](#_ENREF_8) |
|  | 5-14 year olds (deaths per 100,000 population) | 42.6 | [^8^](#_ENREF_8) |
|  | 15-24 year olds (deaths per 100,000 population) | 207.6 | [^8^](#_ENREF_8) |
|  | 25-34 year olds (deaths per 100,000 population) | 369.2 | [^8^](#_ENREF_8) |
|  | 35-44 year olds (deaths per 100,000 population) | 821.6 | [^8^](#_ENREF_8) |
|  | 45-54 year olds (deaths per 100,000 population) | 1,448.5 | [^8^](#_ENREF_8) |
|  | 55-64 year olds (deaths per 100,000 population) | 2,422.1 | [^8^](#_ENREF_8) |
|  | 65+ year olds (deaths per 100,000 population) | 6,182.0 | [^8^](#_ENREF_8) |

**Cultural parameters**

| Percentage of population temporarily away from community of usual residence at any given time | Figure 1 | [^9^](#_ENREF_9) |
| --- | --- | --- |
| Duration of temporary migration (weeks) | 3 [1 - 7]^[[1]](#endnote-1)^ | [^10^](#_ENREF_10) |

**Interaction parameters**

| Probability of close interaction (within the household) between members of the same household | Table 1 | Assumed |
| --- | --- | --- |
| Probability of close interaction (within the household) between usual household member and a temporary visitor | Table 2 | Assumed |
| Probability of close interaction within the community (outside of the household) | Table 3 | Assumed |
| Low interaction probability | [0 – 0.15]^[[2]](#endnote-2)^ | Assumed |
| Moderate interaction probability | [0.15 – 0.3]^2^ | Assumed |
| High interaction probability | [0.3 – 0.45]^2^ | Assumed |
| Very high interaction probability | [0.45 – 0.6]^2^ | Assumed |

**Transmission parameters**

| Community force of infection | [0.127 – 2.460]^[[3]](#endnote-3)^ | Fitted |
| --- | --- | --- |
| Relative probability of community transmission to  household transmission | [0.055 – 0.034]^[[4]](#endnote-4)^ | Fitted |
| Increased infectiousness due to having a dirty face | [1.5 – 2]^2^ | [^11^](#_ENREF_11) |
| Increased susceptibility due to having a dirty face | [1.5 – 2]^2^ | [^11^](#_ENREF_11) |
| Infectivity of an infectious individual | Figure 2 | [^12^](#_ENREF_12) |

**Biological parameters**

| Duration of incubation period (weeks) | 1 [1 - 2]^[[5]](#endnote-5)^ | [^13^](#_ENREF_13) |
| --- | --- | --- |
| Duration of infection in 0-4 year olds (weeks) | 36 [18 - 74]^5^ | [^14^](#_ENREF_14) |
| Duration of infection in 5-14 year olds (weeks) | 19 [12 - 31]^5^ | [^14^](#_ENREF_14) |
| Duration of infection in adults (weeks) | 7 [3 - 19]^5^ | [^14^](#_ENREF_14) |
| Duration of disease in 0-4 year olds (weeks) | 15 [8 - 29]^5^ | [^14^](#_ENREF_14) |
| Duration of disease in 5-14 year olds (weeks) | 8 [5 - 13]^5^ | [^14^](#_ENREF_14) |
| Duration of disease in adults (weeks) | 8 [2 - 27]^5^ | [^14^](#_ENREF_14) |

**Figure 1)** Percentage of population away from community of usual residence at any given time.***
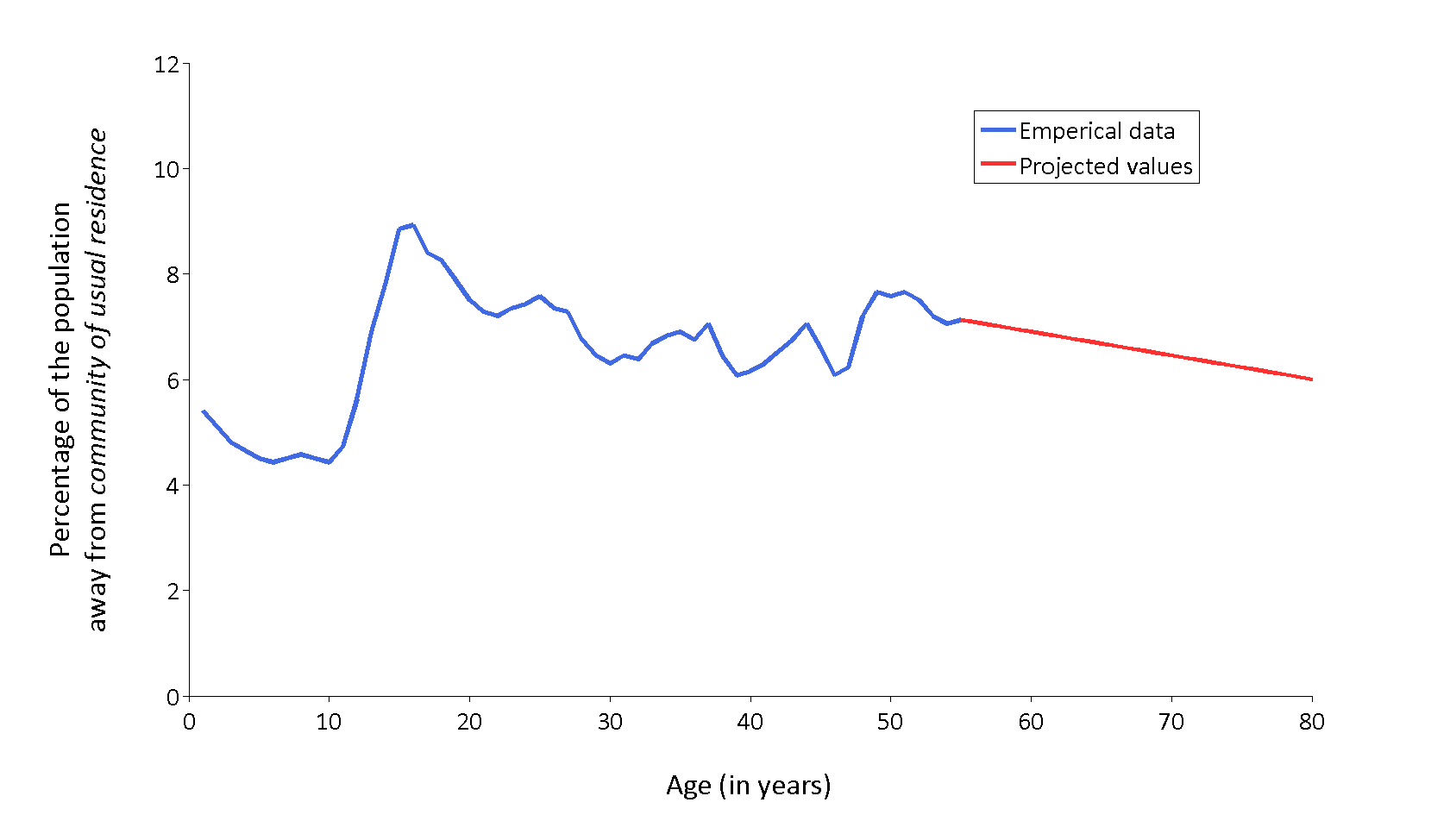
***

**Table 1)** Probability of close household interaction between members of the same household.

|  | 0 - 4 year olds | 5 - 9 year olds | 10 - 14 year olds | 15 + year olds |
| --- | --- | --- | --- | --- |
| 0 - 4 year olds | high | moderate | low | moderate |
| 5 - 9 year olds |  | very high | low | moderate |
| 10 - 14 year olds |  |  | moderate | low |
| 15 + year olds |  |  |  | low |

**Table 2)** Probability of close household interaction between a usual household member and a temporary visitor.

|  | 0 - 4 year olds | 5 - 9 year olds | 10 - 14 year olds | 15 + year olds |
| --- | --- | --- | --- | --- |
| 0 - 4 year olds | moderate | low | low | low |
| 5 - 9 year olds |  | high | low | low |
| 10 - 14 year olds |  |  | low | low |
| 15 + year olds |  |  |  | low |

**Table 3)** Probability of close interaction within the community.

|  | 0 - 4 year olds | 5 - 9 year olds | 10 - 14 year olds | 15 + year olds |
| --- | --- | --- | --- | --- |
| 0 - 4 year olds | moderate | low | low | low |
| 5 - 9 year olds |  | very high | low | low |
| 10 - 14 year olds |  |  | low | low |
| 15 + year olds |  |  |  | low |

**Figure 2)** Relative infectivity of an infectious individual.


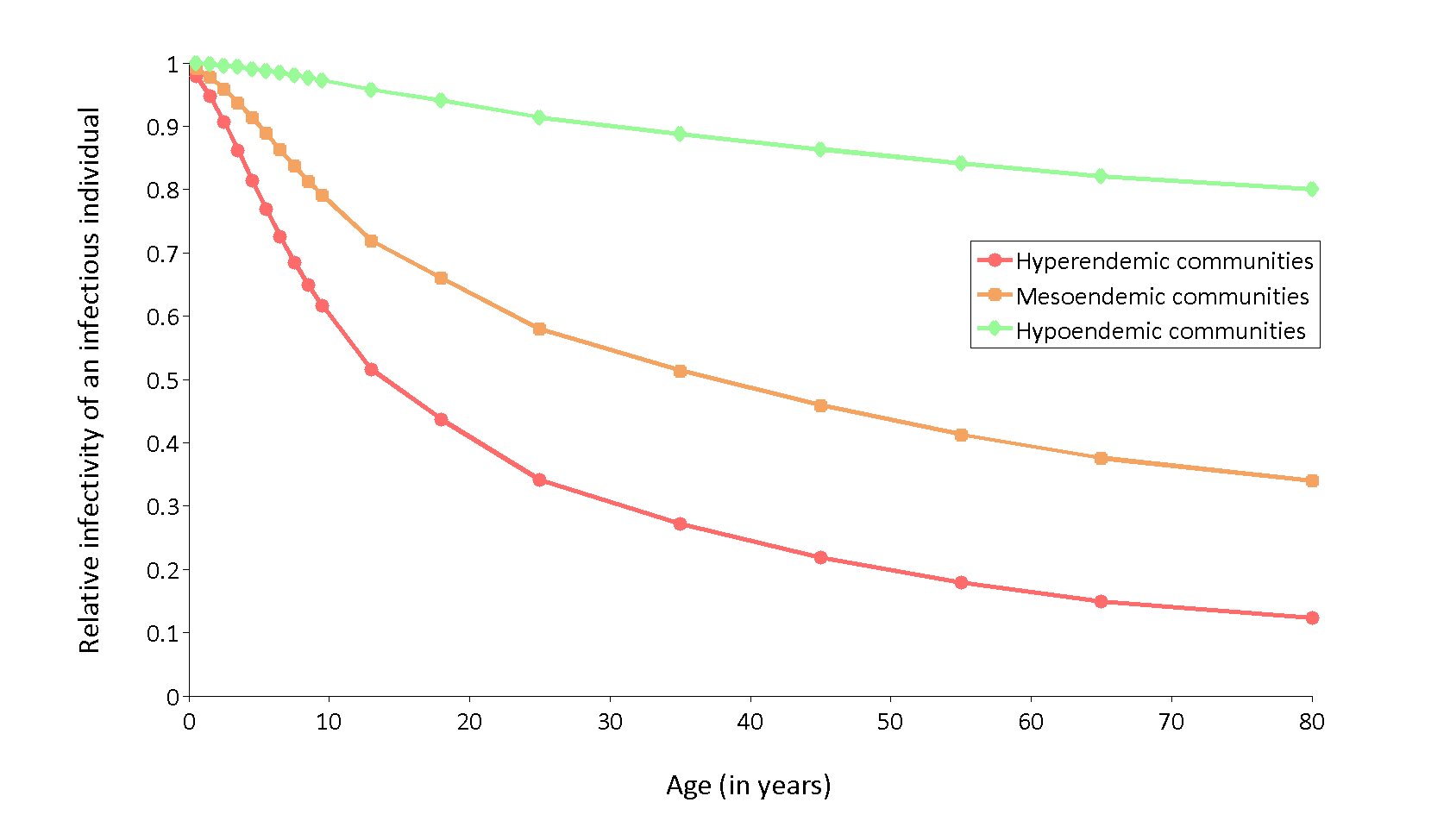


The infectivity of an individual is assumed to be linearly proportional to the bacterial load one carries when infected [^12^](#_ENREF_12). The bacterial load an infected individual harbors is assumed to be dependent upon the number of infections previously suffered [^15^](#_ENREF_15), which is defined as a function of their age and the baseline community disease prevalence.

Bibliography

1. Adams K. Trachoma Surveillance Report 2009: National Trachoma Surveillance and Reporting Unit: Centre for Molecular, Environmental, Genetic and Analytic Epidemiology; 2010.

2. Cowling C. Trachoma Surveillance Report 2010: National Trachoma Surveillance and Reporting Unit: The Kirby Institute; 2011.

3. Lietman TM, Gebre T, Ayele B, Ray KJ, Maher MC, See CW, et al. The epidemiological dynamics of infectious trachoma may facilitate elimination. Epidemics-Neth. 2011; **3**(2): 119-24.

4. Tellis B. Trachoma Surveillance Report 2007: National Trachoma Surveillance and Reporting Unit: Centre for Eye Research Australia; 2008.

5. Tellis B. Trachoma Surveillance Report 2008: National Trachoma Surveillance and Reporting Unit: Centre for Eye Research Australia; 2009.

6. Australian Bureau of Statistics. 2006 Census Community Profiles by Location. 2012 [cited; Available from: <http://www.censusdata.abs.gov.au/ABSNavigation/prenav/ProductSelect?newproducttype=Community+Profiles&btnSelectProduct=Select+Location+%3E&collection=Census&period=2006&areacode=&geography=&method=&productlabel=&producttype=&topic=&navmapdisplayed=true&javascript=true&breadcrumb=P&topholder=0&leftholder=0&currentaction=201&action=104&textversion=false>

7. Australian Bureau of Statistics. Births, Australia. Canberra, ; 2010.

8. Australian Bureau of Statistics. Deaths, Australia. Canberra, ; 2010.

9. Biddle N, Prout S. The geography and demography of Indigenous temporary mobility: an analysis of the 2006 census snapshot. Journal of Population Research. 2009; **26**(4): 305-26.

10. Prout S, Yap M. Indigenous temporary mobilities and service delivery in regional service centres: A West Kimberley case study. Canberra: Centre for Aboriginal Economic Policy Research; 2010.

11. Taylor HR. Trachoma: A blinding scourge from the bronze age to the twenty-first century: Centre for Eye Research Australia; 2008.

12. Gambhir M, Basanez M-G, Blake IM, Grassly NC. Modelling trachoma for control programmes. Adv Exp Med Biol. 2010; **673**: 141-56.

13. Jawetz E, Rose L, Hanna L, Thygeson P. Experimental inclusion conjunctivitis in man: measurements of infectivity and resistance. JAMA : the journal of the American Medical Association. 1965; **194**(6): 620-32.

14. Grassly NC, Ward ME, Ferris S, Mabey DC, Bailey RL. The natural history of trachoma infection and disease in a Gambian cohort with frequent follow-up. PLoS Negl Trop Dis. 2008; **2**(12): e341.

15. West ES, Munoz B, Mkocha H, Holland MJ, Aguirre A, Solomon AW, et al. Mass treatment and the effect on the load of Chlamydia trachomatis infection in a trachoma-hyperendemic community. Investigative ophthalmology & visual science. 2005; **46**(1): 83-7.

1. Mean [Range] [↑](#endnote-ref-1)
2. Range of the uniform distributions from which Latin hypercube samples were taken. [↑](#endnote-ref-2)
3. Range obtained from fitting to annual community-level age-stratified trachoma disease prevalence data from the realistic range of values (0, 10), with values being unique for each community. [↑](#endnote-ref-3)
4. Range obtained from fitting to annual community-level age-stratified trachoma disease prevalence data from the realistic range of values (0, 1). [↑](#endnote-ref-4)
5. Median [Range] [↑](#endnote-ref-5)
