# Supplementary material for: Control of Trachoma in Australia: A Model Based Evaluation of Current Interventions
Source: PLoS Negl Trop Dis. 2015 Apr 10;9(4):e0003474. doi: 10.1371/journal.pntd.0003474 (PMC4393231; doi:10.1371/journal.pntd.0003474)
Supplement: S2 Table — (DOCX) [file pntd.0003474.s002.docx]

S2 Table. Extrapolating current intervention trends.

In order to quantify the likelihood that Australia’s current control effort will achieve the target of national trachoma elimination by 2020, an intervention strategy was compiled that incorporates extrapolations of the observed trends in outcomes from control efforts of previous screening and treatment events. To construct this projected strategy, national surveillance data from 2007 to 2011 was analysed for time-varying trends of control variables such as screening and treatment coverage, screening and treatment synchronicity, and the prevalence of facial cleanliness in children. The community-specific trends ascertained from the data were combined to generate the expected future intervention effort should the national trachoma control guidelines remain unaltered. Due to a large amount of noise in the observed variables, a series of constant values were generated to best describe the likely future control effort. Table 1 details the values of these constants.

**Table 1)** Extrapolation of current intervention trends.

| Modelled region | | Predominantly hyperendemic region | Predominantly mesoendemic region | Predominantly hypoendemic region |
| --- | --- | --- | --- | --- |
| Screening coverage (%) | 1-4 year olds | 12.3 | 13.5 | 18.6 |
|  | 5-9 year olds | 81.6 | 70.5 | 78.9 |
|  | 10-14 year olds | 61.7 | 54.9 | 61.4 |
| Treatment coverage (%) | | 84.8 | 87.6 | 86.1 |
| Clean Face prevalence (%) | 1-4 year olds | 33.3 | 75.2 | 81.7 |
|  | 5-9 year olds | 60.0 | 83.6 | 87.1 |
|  | 10-14 year olds | 86.0 | 94.0 | 95.8 |
| Duration of treatment session | | 1–4 Weeks | 1-3 Weeks | 1-3 Weeks |
| Delay between screening and treatment | | 0-9 Weeks | 0-2 Weeks | 1-9 Weeks |
