# Supplementary material for: Control of Trachoma in Australia: A Model Based Evaluation of Current Interventions
Source: PLoS Negl Trop Dis. 2015 Apr 10;9(4):e0003474. doi: 10.1371/journal.pntd.0003474 (PMC4393231; doi:10.1371/journal.pntd.0003474)
Supplement: S3 Table — (DOCX) [file pntd.0003474.s003.docx]

S3 Table. Current and proposed guidelines.

**Table 1)** The current strategy for trachoma control in Australia.

| Prevalence of active disease in 1-9 year olds | Treatment procedure | Screening procedure |
| --- | --- | --- |
| $\geq$ 10%  with no obvious household clustering | Treat all index cases and  their household contacts.  Also treat all 1 – 14 year olds in the community. | Re-screen community  in 12 months |
| $\geq$ 10%  with obvious household clustering | Treat all index cases and  their household contacts. | Re-screen community  in 12 months |
| $\geq$ 5% but $<$ 10% | Treat all index cases and  their household contacts. | Re-screen community  in 12 months |
| $<$ 5% | Treat all index cases and  their household contacts. | Re-screen community  in 12 months |
| $<$ 5%  for 5 consecutive years | Treat all index cases and  their household contacts. | Cease screening |

**Table 2)** The target intensity for the implementation of the current national trachoma control guidelines.

| Activity | Trachoma Project Agreement Targets |
| --- | --- |
| Trachoma Screening | At least 80% of 5-9 year old children residing in ‘at-risk’ communities are screened for active trachoma over a 12 month period |
| Active Trachoma Treatment | At least 90% of children with active trachoma and at least 90% of their household contacts are treated with antibiotics |
|  | Antibiotic treatment of index cases and their household contacts within each community are completed within a two week period |
| Facial Cleanliness Promotion | The concept of a ‘clean face’ is actively promoted and a facial cleanliness prevalence of 70% is found amongst children screened for trachoma |

**Table 3)** The proposed future strategy for trachoma control in Australia.

| Prevalence of active disease in 5-9 year olds | Treatment procedure | Treatment frequency | Screening frequency |
| --- | --- | --- | --- |
| $\geq$ 20% | Mass Drug Administration | 0, 6, 12, 18 and 24 months  after initial screening | Re-screen community 36 months from initial screening |
| $\geq$ 5% but $<$ 20% | Mass Drug Administration | 0, 12 and 24 months  after initial screening | Re-screen community 36 months from initial screening |
| $<$ 5% | Treat all index cases and  their household contacts | Once at 0 months and then again if trachoma is found upon subsequent screening | Re-screen community 12, 36 and 50 months from initial screening |

**Table 4)** The target intensity for the implementation of the proposed national trachoma control strategy.

| Activity | Trachoma Project Agreement Targets |
| --- | --- |
| Trachoma Screening | At least 85% of 5-9 year old children residing in ‘at-risk’ communities are screened for active trachoma over a 12 month period |
| Active Trachoma Treatment | 100% of children with active trachoma and at least 85% of their household contacts are treated with antibiotics |
|  | Antibiotic treatment of index cases and their household contacts within each community are completed within a two week period |
| Facial Cleanliness Promotion | The concept of a ‘clean face’ is actively promoted and a facial cleanliness prevalence of 85% is found amongst children screened for trachoma |
