# Supplementary material for: Control of Trachoma in Australia: A Model Based Evaluation of Current Interventions
Source: PLoS Negl Trop Dis. 2015 Apr 10;9(4):e0003474. doi: 10.1371/journal.pntd.0003474 (PMC4393231; doi:10.1371/journal.pntd.0003474)
Supplement: S1 Text — (DOCX) [file pntd.0003474.s004.docx]

S1 Text. Underlying model assumptions.

To describe the trachoma epidemic in remote Australia, an agent-based stochastic transmission simulation model was constructed [^1^](#_ENREF_1). The model was used to forecast the expected epidemic trends of trachoma disease prevalence in three representative regions of rural Australia under a range of different control initiatives. The three regions to be modelled were selected based on the level of surveillance data that have been extracted from the region, and the mean endemicity of the communities within the region. The model simulates the transmission of *Chlamydia trachomatis* infection among Aboriginal people in selected ‘at-risk’ communities within each of these remote regions separately. The contraction of infection and consequent development of disease are recorded and tracked at the individual level. The main assumptions of the model are summarised in the following subsections.

### Demographic assumptions

Each modelled individual is a member of a community encompassed by the region, and is also a resident of a household within their community. An individual is attributed a propensity to temporarily migrate from their usual abode in their home community, which is dependent upon their age [^2^](#_ENREF_2). If it is determined that an individual is to temporarily migrate from their home community at a given time, the model specifies the destination community and household that the individual is to travel to. Each community that is represented in the model is coupled with neighbouring communities, such that the likelihood of an individual migrating to a given community is dictated primarily by its proximity to the individual’s home community. This likelihood is also influenced by qualitative information from expert sources.

The probability of death is dependent only upon age and occurs irrespective of trachoma state, whilst the likelihood of a newborn entering the population and into a given household is dependent upon the number of adults permanently living within the residence.

### Transmission assumptions

The transmission of *Chlamydia trachomatis* infection and the consequent development of disease are monitored in the model at the individual level. That is, the probability of infection transmission between two individuals is calculated and a probabilistic operation is executed to determine whether transmission should occur at a given time point. The probability of infection transmission between the two individuals is influenced by the following factors and personal characteristics:

- The likelihood of close interaction between the two individuals - which is dependent upon their ages and if they are residents of the same household ^[3](#_ENREF_3)^.
- The infectivity of the infected individual – which is based on the expected number of previous infections [^4^](#_ENREF_4).
- The facial cleanliness of the susceptible individual [^5^](#_ENREF_5).
- The facial cleanliness of the infectious individual [^5^](#_ENREF_5).

Two distinct platforms for interaction, and thus transmission potential, are considered in the model. The primary setting for transmission possibility is the household ^[6](#_ENREF_6)^, while the secondary setting in which transmission can occur is the community. Consequently, it is necessary for the probability of transmission between two individuals to also be influenced, on both the household and community level, by a multiplicative factor known as a *force of infection*. The difference in value of the force of infection factors for the household and the community settings represents the unequal efficiency of infection transmission between people within, and outside of, the household. The force of infection factors, which are distinct for each community, absorb any external factors that are not explicitly considered in the model, such as the environmental conditions of a community or the volume of flies. The numerical value of each multiplicative factor is determined through the calibration process which matches model outputs with available empirical data.

### Interaction assumptions

As previously described, interaction between individuals may occur within the household or within the community. The probability of an interaction that may facilitate the transmission of *Chlamydia trachomatis* (that is, hand-to-eye contact or face-to-face contact) between two individuals is determined by their ages [^5^](#_ENREF_5). This probability of close, potential transmission inducing, interaction between two people differs for each of the following scenarios.

- The interaction occurs within the household, and the two individuals are usual residents of the same household.
- The interaction occurs within the household, and one or both of the individuals are temporarily residing in the household.
- The interaction occurs outside of the household but within the community.

### Force of infection equations

The probability of a susceptible individual $i$ in community $c$ contracting infection from an infected individual $j$ within the *community* setting is governed by the equation

$$\lambda_{c}=\beta_{c}{\cdot\omega_{c}\left[ \hat{a}_{i}, \hat{a}_{j} \right]\cdot\varphi}_{a_{j}}\cdot f\left( i \right)\cdot f\left( j \right)$$

where

- $a_{k}$ is the age and $\hat{a}_{k}$ is the age group (0-4, 5-9, 10-14 or 15+ years) of an individual $k$.
- $\beta_{c}$ is the calibrated community transmission coefficient for an interaction in community $c$.
- $\omega_{c}$ is the community level contact matrix (see parameter table ***d)*** below), and $\omega_{c}\left[ \hat{a}_{i}, \hat{a}_{j} \right]$ is the $\left( \hat{a}_{i}, \hat{a}_{j} \right)$^th^ entry of the matrix.
- $\varphi_{a_{k}}$ is the infectivity of an infectious individual $k$.
- $f\left( k \right)=\left\{ \begin{aligned} 1, &\text{if individual }k\text{ has a dirty face} \\ 1-\gamma, \text{if individual }k\text{ has a clean face} \end{aligned} \right.$
- $\gamma$ is the reduction in transmissibility due to facial cleanliness.

The probability of a susceptible individual $i$ in community $c$ contracting infection from an infected *fellow household resident* $j$ within the *household* setting is governed by the equation

$$\lambda_{h}=\beta_{h_{c}}{\cdot\omega_{h}\left[ \hat{a}_{i}, \hat{a}_{j} \right]\cdot\varphi}_{a_{j}}\cdot f\left( i \right)\cdot f\left( j \right)$$

where

- $\beta_{h_{c}}$ is the calibrated household transmission coefficient for an interaction within a household in community $c$.
- $\omega_{h}$ is the household level contact matrix (see parameter table ***b)*** below), and $\omega_{h}\left[ \hat{a}_{i}, \hat{a}_{j} \right]$ is the $\left( \hat{a}_{i}, \hat{a}_{j} \right)$^th^ entry of the matrix.

The probability of a susceptible individual $i$ in community $c$ contracting infection from an infected individual $j$ *temporarily residing* in the same household is governed by the equation

$$\lambda_{t}=\beta_{h_{c}}{\cdot\omega_{t}\left[ \hat{a}_{i}, \hat{a}_{j} \right]\cdot\varphi}_{a_{j}}\cdot f\left( i \right)\cdot f\left( j \right)$$

where

- $\omega_{t}$ is the household level contact matrix for temporary residents (see parameter

table ***c)*** below), and $\omega_{t}\left[ \hat{a}_{i}, \hat{a}_{j} \right]$ is the $\left( \hat{a}_{i}, \hat{a}_{j} \right)$^th^ entry of the matrix.

Bibliography

1. Keeling MJ, Rohani P. Modeling Infectious Diseases in Humans and Animals: Princeton University Press; 2007.

2. Biddle N, Prout S. The geography and demography of Indigenous temporary mobility: an analysis of the 2006 census snapshot. Journal of Population Research. 2009; **26**(4): 305-26.

3. Blake IM, Burton MJ, Solomon AW, West SK, Basanez M-G, Gambhir M, et al. Targeting antibiotics to households for trachoma control. PLoS Negl Trop Dis. 2010; **4**(11): e862.

4. Gambhir M, Basanez M-G, Blake IM, Grassly NC. Modelling trachoma for control programmes. Adv Exp Med Biol. 2010; **673**: 141-56.

5. Taylor HR. Trachoma: A blinding scourge from the bronze age to the twenty-first century: Centre for Eye Research Australia; 2008.

6. Blake IM, Burton MJ, Bailey RL, Solomon AW, West S, Munoz B, et al. Estimating household and community transmission of ocular Chlamydia trachomatis. PLoS Negl Trop Dis. 2009; **3**(3): e401.
