# Supplementary material for: Control of Trachoma in Australia: A Model Based Evaluation of Current Interventions
Source: PLoS Negl Trop Dis. 2015 Apr 10;9(4):e0003474. doi: 10.1371/journal.pntd.0003474 (PMC4393231; doi:10.1371/journal.pntd.0003474)
Supplement: S1 File — (DOCX) [file pntd.0003474.s005.docx]

S1 File. Calibration process and sensitivity analysis.

**Calibration process**

The model was independently calibrated to empirical age-stratified community-level disease prevalence data from three de-identified Australian regions through a Monte Carlo filtering process. The timing and intensity data of screening and treatment activities as described by the data available in the national trachoma database, which is made available in summarised form though annual national trachoma surveillance reports [[1-4](#_ENREF_1)], were entered directly into the model. The community-level age-stratified prevalence of facial cleanliness amongst children was also directly entered into the model as described by the national data set.

The *interaction* and *transmission* parameters (as described in the parameter table above) that are components of the force of infection calculation were sampled through uniform-distribution Latin-hypercube sampling to form 1,000 unique parameter sets that satisfy the condition that community-level prevalence at the time of community screening is no greater than 150% or no less than 50% of the annual prevalence estimate for the respective community. The community-specific force of infection parameters were varied for each community such that this condition was satisfied; the other parameters were constant across each community within a region. The *biological* parameters of the model (as described in the previous section) were not calibrated, but are sampled from the specified distributions when an individual initiates the respective disease stage.

**Sensitivity analysis**

Both a linear regression analysis and a partial-rank-correlation-coefficient analysis were performed on the 1,000 calibrated parameter sets and the corresponding model output chosen was 5-9 year old disease prevalence in 2020. These analyses indicate that the model results are most heavily influenced by the household and community level transmission parameters (*beta household* and *beta community*, respectively) and the assumed reduction in transmission due to facial cleanliness (*gamma*). However, no parameters were very highly influential in solely driving trends, as indicated by correlation coefficients less than 0.5.

**Figure 1)** Linear regression analysis.


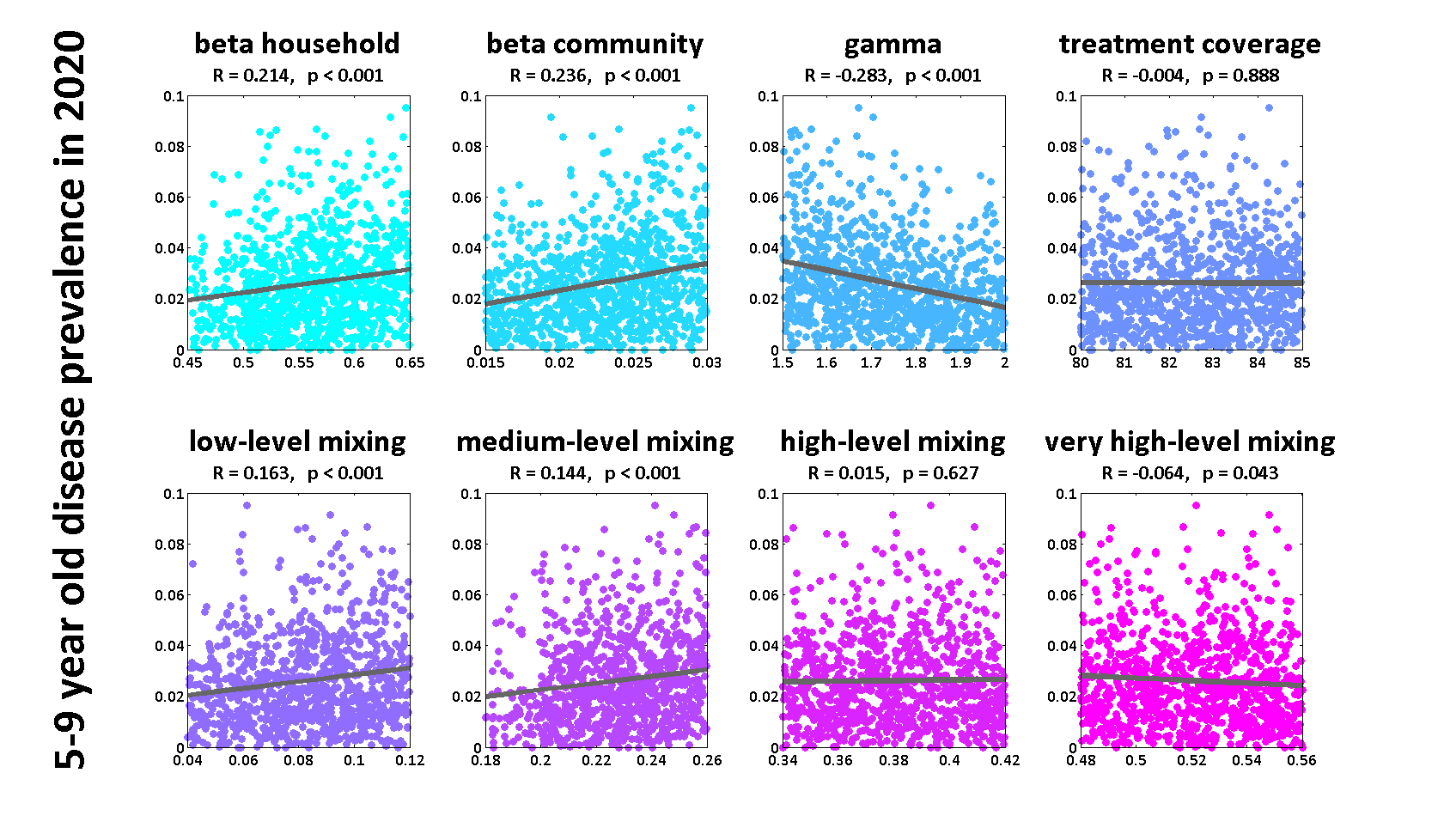


**Figure 2)** Partial-rank-correlation-coefficient analysis.


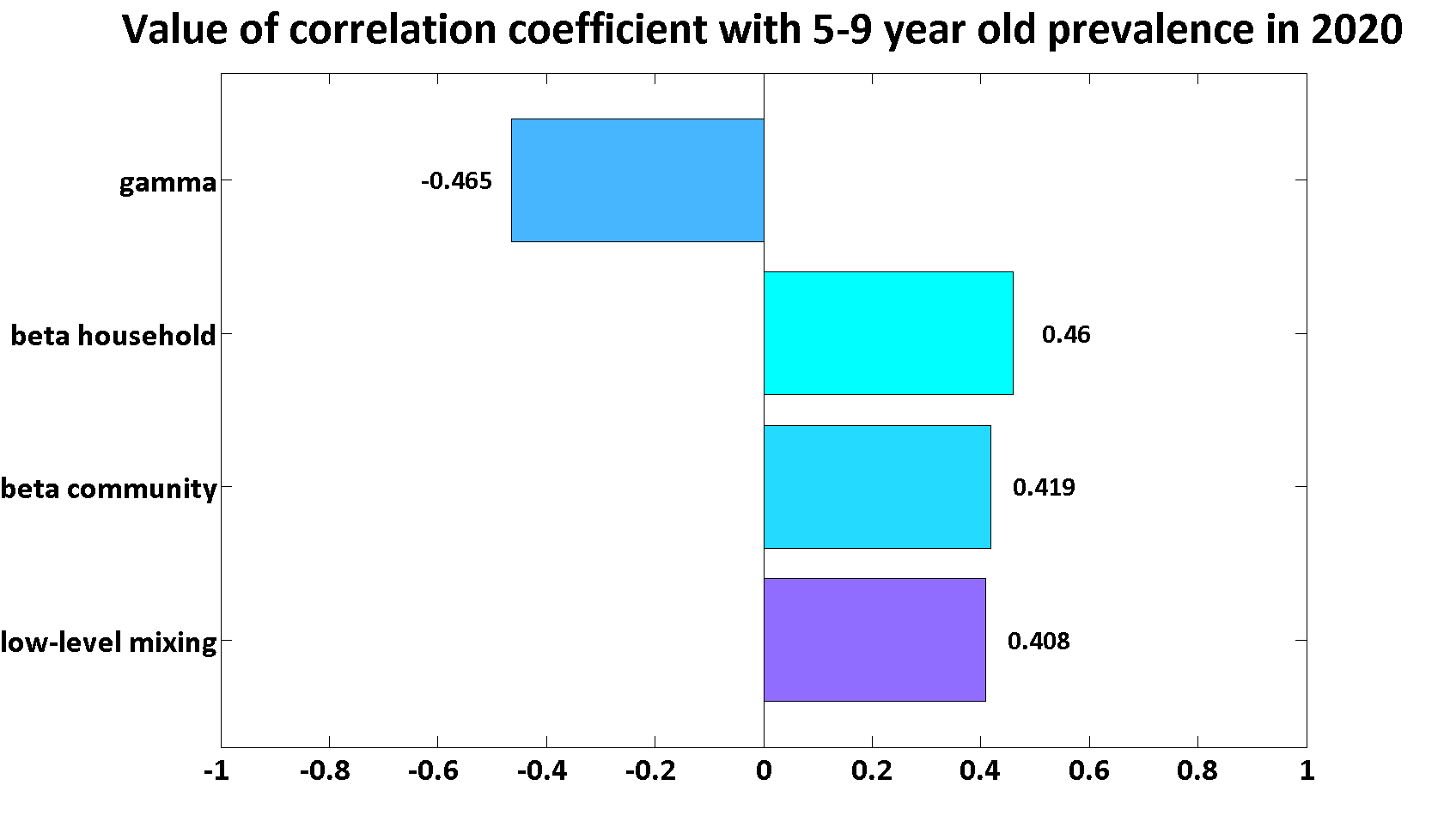


Bibliography

1. Tellis B (2008) Trachoma Surveillance Report 2007: National Trachoma Surveillance and Reporting Unit. Centre for Eye Research Australia.

2. Tellis B (2009) Trachoma Surveillance Report 2008: National Trachoma Surveillance and Reporting Unit. Centre for Eye Research Australia.

3. Adams K (2010) Trachoma Surveillance Report 2009: National Trachoma Surveillance and Reporting Unit. Centre for Molecular, Environmental, Genetic and Analytic Epidemiology.

4. Cowling C (2011) Trachoma Surveillance Report 2010: National Trachoma Surveillance and Reporting Unit. The Kirby Institute.
